# Small Cell Carcinoma of the Ovary, Hypercalcemic Type (SCCOHT): Patient Characteristics, Treatment, and Outcome—A Systematic Review

**DOI:** 10.3390/cancers15153794

**Published:** 2023-07-26

**Authors:** Francis S. P. L. Wens, Caroline C. C. Hulsker, Marta Fiocco, József Zsiros, Stephanie E. Smetsers, Ronald R. de Krijger, Alida F. W. van der Steeg, Ronald P. Zweemer, Inge O. Baas, Eva Maria Roes, Leendert H. J. Looijenga, Cornelis G. Gerestein, Annelies M. C. Mavinkurve-Groothuis

**Affiliations:** 1 Princess Máxima Center for Pediatric Oncology, 3584 CS Utrecht, The Netherlands; franciswens1996@hotmail.com (F.S.P.L.W.); c.c.c.hulsker@prinsesmaximacentrum.nl (C.C.C.H.); m.f.fiocco@prinsesmaximacentrum.nl (M.F.); j.zsiros@prinsesmaximacentrum.nl (J.Z.); s.e.smetsers-3@prinsesmaximacentrum.nl (S.E.S.); r.r.dekrijger-2@prinsesmaximacentrum.nl (R.R.d.K.); a.f.w.vandersteeg@prinsesmaximacentrum.nl (A.F.W.v.d.S.); l.looijenga@prinsesmaximacentrum.nl (L.H.J.L.); 2 Mathematical Institute, Leiden University, 2311 EZ Leiden, The Netherlands; 3 Biomedical Data Science Department, Section Medical Statistics, Leiden University Medical Center, 2333 ZA Leiden, The Netherlands; 4 Department of Pathology, University Medical Center Utrecht, 3584 CG Utrecht, The Netherlands; 5 Department of Gynecologic Oncology, Division of Imaging and Oncology, University Medical Center Utrecht, 3584 CG Utrecht, The Netherlands; r.zweemer@umcutrecht.nl (R.P.Z.); c.g.gerestein-2@umcutrecht.nl (C.G.G.); 6 Department of Medical Oncology, University Medical Center Utrecht, 3584 CG Utrecht, The Netherlands; i.o.baas-3@umcutrecht.nl; 7 Department of Gynecologic Oncology, Erasmus Medical Center Cancer Institute, 3015 GD Rotterdam, The Netherlands; e.roes@erasmusmc.nl

**Keywords:** small-cell carcinoma of the ovary, SCCOHT, hypercalcemia, SMARCA4, ovarian tumor, ovarian carcinoma

## Abstract

**Simple Summary:**

Small-cell carcinoma of the ovary, hypercalcemic type (SCCOHT) is a rare and aggressive ovarian cancer with a poor prognosis, and information on adequate treatment for this cancer is lacking. However, since the discovery of mutations in the SMARCA4 gene in 2014, SCCOHT has become the subject of extensive investigation. With this systematic review, we aim to generate an overview of all reported patients with SCCOHT from 1990 onwards, describing the clinical presentation, genetic characteristics, treatment, and outcome. Harmonization and international collaboration to obtain high-quality data on diagnostic investigations, treatment, and outcome are warranted to be able to develop international treatment guidelines to improve the survival chances of young women with SCCOHT.

**Abstract:**

Background: Small-cell carcinoma of the ovary, hypercalcemic type (SCCOHT) is a rare aggressive ovarian malignancy mainly affecting children, adolescents, and young adults. Since the discovery of mutations in the SMARCA4 gene in 2014, SCCOHT has become the subject of extensive investigation. However, international uniform treatment guidelines for SCCOHT are lacking and the outcome remains poor. The aim of this systematic review is to generate an overview of all reported patients with SCCOHT from 1990 onwards, describing the clinical presentation, genetic characteristics, treatment, and outcome. Methods: A systematic search was performed in the databases Embase, Medline, Web of Science, and Cochrane for studies that focus on SCCOHT. Patient characteristics and treatment data were extracted from the included studies. Survival was estimated using Kaplan–Meier’s methodology. To assess the difference between survival, the log-rank test was used. To quantify the effect of the FIGO stage, the Cox proportional hazard regression model was estimated. The chi-squared test was used to study the association between the FIGO stage and the surgical procedures. Results: Sixty-seven studies describing a total of 306 patients were included. The median patient age was 25 years (range 1–60 years). The patients mostly presented with non-specific symptoms such as abdominal pain and sometimes showed hypercalcemia and elevated CA-125. A great diversity in the diagnostic work-up and therapeutic approaches was reported. The chemotherapy regimens were very diverse, all containing a platinum-based (cisplatin or carboplatin) backbone. Survival was strongly associated with the FIGO stage at diagnosis. Conclusions: SCCOHT is a rare and aggressive ovarian cancer, with a poor prognosis, and information on adequate treatment for this cancer is lacking. The testing of mutations in SMARCA4 is crucial for an accurate diagnosis and may lead to new treatment options. Harmonization and international collaboration to obtain high-quality data on diagnostic investigations, treatment, and outcome are warranted to be able to develop international treatment guidelines to improve the survival chances of young women with SCCOHT.

## 1. Introduction

Small-cell carcinoma of the ovary, hypercalcemic type (SCCOHT) is a rare aggressive ovarian malignancy and is associated with hypercalcemia in two-thirds of cases. SCCOHT accounts for less than 0.01% of ovarian neoplasms with a peak incidence in young adulthood (range 0–55 years with a median of approximately 24 years) [1,2].

In contrast to most undifferentiated ovarian carcinomas, which are composed of large cells with abundant cytoplasm, this tumor is typically composed of small cells [1]. SCCOHT was first considered a highly malignant variant of granulosa-cell tumor because of the presence of follicles [1]. However, in the 2014 World Health Organization (WHO) Classification of Tumours of Female Reproductive Organs, SCCOHT was classified as a miscellaneous neoplasm [3]. Additionally, SCCOHT has been noted to share similarities with rhabdoid tumors. Both SCCOHT and rhabdoid tumors show mutations in members of the SWI/SNF complex and demonstrate poor response to conventional therapy [4].

Tumors are staged according to the FIGO guidelines for ovarian cancer. The FIGO stage remains the mainstay in the assessment of prognosis, with FIGO stage IA disease having the most favorable prognosis with a five-year overall survival (OS) of 33% [5,6]. Other potentially favorable prognostic features include the following: age > 30 years, normal preoperative calcium level, tumor size of up to 10 cm, and absence of large cells [5]. Current knowledge is based on small case series and case reports. The expected survival regardless of the tumor stage shows an estimated one-year survival rate of 50% and a five-year survival rate of less than 10% [7].

Although various treatment approaches have been proposed, there is no international consensus on medical therapy and surveillance. A multimodal approach including radical cytoreductive surgery, platinum-based chemotherapy, whole-abdomen radiotherapy, and high-dose chemotherapy with autologous stem cell transplantation (HDC ASCT) is often proposed [8].

The occurrence of SCCOHT in several members of the same family raised the hypothesis that some cases could have a genetic background [9]. In 2014, Witkowski et al. discovered that germline and somatic SMARCA4 mutations are causally related to SCCOHT in approximately 95% of the patients, which led to improvements in genetic counseling [10]. Genetic testing for at-risk relatives is recommended since such variants are inherited in an autosomal dominant manner. Surveillance for at-risk family members remains controversial given the lack of proven efficacy and the potential risks including a false sense of security, the risk of false-positive screens, and the potential exclusion of effective risk-reducing surgery [11].

The aim of this study is to generate an overview of the current knowledge on the incidence, treatment, genetic characteristics, clinical presentation, and survival outcomes of SCCOHT in order to acquire an input that can be used as a starting point to set up a prospective (international) database that will eventually lead to treatment recommendations.

## 2. Materials and Methods

### 2.1. Search Strategy and Study Selection

This systematic review was performed according to the PRISMA Statement [12]. A systematic search was conducted for available published articles from 1990 to 12 December 2022 in the databases Embase, Medline, Web of Science, and Cochrane. These databases were searched for studies that focused on ovarian malignancies, particularly SCCOHT. Supplementary Table S1 provides the full search strategy. We chose to perform a systematic search from 1990 onwards, since Young et al. published a comprehensive review in 1994 that included all cases from before 1990 [5]. After eliminating duplicates, three authors (A.M., C.H., and F.W.) independently screened all remaining publications for potentially eligible studies based on title and abstract. After comparison and consensus of the authors on the potentially relevant articles, they were screened on the eligibility criteria based on their full text. Finally, the Guidelines for Snowballing in Systematic Literature were used to conduct a manual search of references [13].

### 2.2. Eligibility Criteria

Articles were considered eligible when the clinical presentations, treatments, and outcomes of patients with SCCOHT were described. Moreover, we decided to only include studies that described a complete follow-up. Since SCCOHT is a rare malignancy, single case reports were included to minimize the risk of missing relevant studies. The included articles had to be available as full text in English. Patients were not included if they died before start of treatment. Reviews, conference abstracts, and animal studies were excluded as well. The same applies to studies that reported on the large cell variant of SCCOHT based on the different morphology and a lack of genetically verified SMARCA4 mutations.

### 2.3. Quality Assessment

The individual quality of the included studies was assessed using the checklist of “The Strengthening the Reporting of Observational Studies in Epidemiology” (STROBE) Statement. This checklist specifies how observational research should be reported [14]. Supplementary Table S2 provides the full STROBE checklist.

### 2.4. Data Collection and Data Items

The following data were collected from the included studies: author and title; year of publication; number of patients; age at diagnosis; symptoms; palpable mass at physical examination; imaging modalities used at diagnosis; tumor localization; presence of hypercalcemia (not further defined because of heterogeneous reporting in the various publications); epithelial tumor markers such as cancer antigen 125 (CA-125), cancer antigen 19-9 (CA 19-9), cancer antigen 15-3 (CA 15-3), cancer antigen 72-4 (CA 72-4), other tumor markers such as Beta-Human Chorionic Gonadotropin (Beta-HCG), Alpha-fetoprotein (AFP), and Inhibin B; surgical procedure; the number of operations; FIGO-stage; chemotherapy; chemotherapy cycles; HDC ASCT; radiotherapy; complications; genetics; recurrence; median follow-up time; and survival. For several patients, the FIGO stage was unknown. These stages were assigned, where possible, in consultation by three authors (C.G., C.H., and F.W.) using the FIGO guidelines, if adequate information on surgery and pathology was available [6]. Besides collecting patient diagnostics and treatment items, we also screened the articles for suggestions for future targeted therapies.

### 2.5. Statistical Analyses

SPSS software 28.0 for Windows was used for data management and statistical analysis. Overall survival (OS) from diagnosis until dead of disease or last contact was estimated using Kaplan–Meier’s methodology. The log-rank test was used to assess the difference between survival. To quantify the effect of the FIGO stage as a prognostic factor for survival, the Cox proportional hazard regression model was used. The chi-squared test was applied to study the association between the FIGO stage and the surgical procedures. FIGO stages II, III, and IV were put together in a single category and compared with FIGO I. A *p*-value below 0.05 (*p* < 0.05) was considered significant.

## 3. Results

### 3.1. Search and Selection

**Figure 1:**
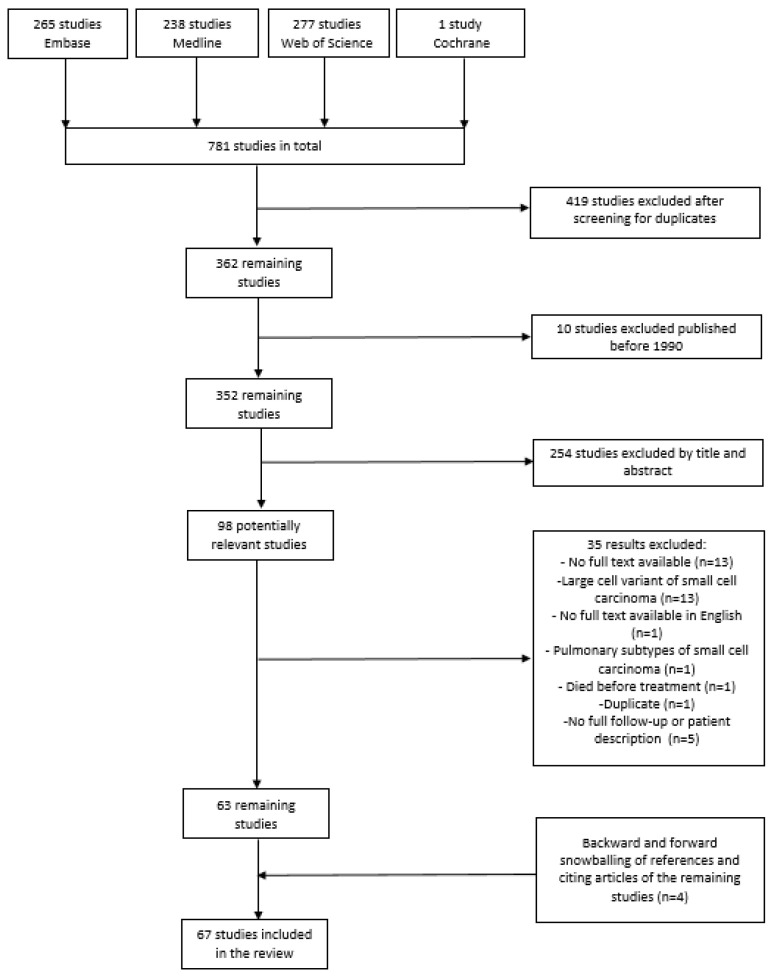
Flowchart of included and excluded articles during the search process.

The initial database searches identified 814 published articles. After removing the duplicates and articles published before 1990, and after elimination by title and abstract and full-text review, 67 studies were included in this systematic review (Table S3) [1,2,5,15,16,17,18,19,20,21,22,23,24,25,26,27,28,29,30,31,32,33,34,35,36,37,38,39,40,41,42,43,44,45,46,47,48,49,50,51,52,53,54,55,56,57,58,59,60,61,62,63,64,65,66,67,68,69,70,71,72,73,74,75,76,77,78]. The study selection process, based on the PRISMA scheme, is depicted in Figure 1.

### 3.2. Quality Assessment

Supplementary Table S2 shows a summary of the quality assessment of the 67 included articles using the STROBE checklist.

### 3.3. Patient Characteristics

A total of 306 patients are described. The characteristics of the included patients are presented in Table 1. The median age is 25 years (range 1–60 years). 

The clinical presentations varied from symptoms associated with a pelvic mass to general symptoms including nausea, fatigue, weight loss, and constipation. Most patients experienced more than one symptom. Abdominal pain was the most frequently mentioned symptom (*n* = 172, 40%), followed by abdominal swelling (*n* = 87, 20%). The other 40% consisted of general and other symptoms. Hypercalcemia was reported in approximately 50% of the cases. A total of 140 tumors were FIGO stage I (45.7%), 19 were stage II (6.2%), 123 were stage III (40.1%), 23 were stage IV (7.5%), and 1 tumor was of unknown stage.

The epithelial marker CA-125, available in 54 cases, was elevated in 43 patients (80%). In some studies, Beta-HCG, AFP, and Inhibin B were determined. In these studies, these markers were within the normal range, as is expected for SCCOHT. In all patients, the diagnosis was established via pathology. Since the discovery of potential underlying SMARCA4 mutations in 2014, 24 tumors were reported to have SMARCA4 loss. We could not find whether a mutation analysis was performed in the other patients.

### 3.4. Treatment

#### 3.4.1. Surgery

Three hundred and three of the included patients underwent surgery alone or surgery in combination with other treatment modalities (Table 2). The surgical procedures described in the different studies were very heterogeneous. The most common surgical procedures were unilateral salpingo-oophorectomy (USO) and total abdominal hysterectomy with bilateral salpingo-oophorectomy (TAHBSO). For patients whose type of surgery was known, a USO was reported in 119 cases and a TAHBSO was reported in 65 cases. USO as a first-stage procedure, followed by a complete hysterectomy and a contralateral salpingo-oophorectomy, was reported in 28 cases in the case of recurrence or after the initial diagnostic surgery. Supplementary Figure S1 shows no significant relationship between the type of surgery and the FIGO stage.

Three patients did not have surgery and fifty-six patients had surgery once. A number of patients underwent multiple surgeries; 28 patients underwent surgery twice, 4 patients underwent surgery three times, and 1 patient had surgery four times. For 214 patients, the number of surgical procedures remained unclear.

#### 3.4.2. Chemotherapy and HDC with ASCT

The first- and second-line chemotherapy regimens were very diverse; however, they were all platinum based with either cisplatin or carboplatin as a backbone, with PAVEP (cisplatin, doxorubicin, cyclophosphamide, and etoposide) and VPCBAE (vinblastine, cisplatin, cyclophosphamide, bleomycin, doxorubicin, and etoposide) as the most frequently used treatment regimens. In 46 cases, standard chemotherapy was followed by HDC ASCT. A variety of more recent chemotherapeutic agents were addressed in the third line, including Temozolomide, Nedaplatin, and Bevacizumab. The reported treatment-induced toxicities were neutropenia, leukocytopenia, thrombocytopenia, anemia, sepsis, mucositis, nausea, vomiting, fatigue, diarrhea, dehydration, renal failure, and ototoxicity (Table 3).

#### 3.4.3. Radiotherapy

At least 45 patients underwent radiotherapy, consistently in combination with other treatment modalities. Although prior studies have recommended radiotherapy in the cases of advanced disease at diagnosis, no relationship between radiotherapy and FIGO stage was identified. Irradiated areas included the whole abdomen, pelvis, para-aortic and pelvic lymph nodes, whole brain, and retroperitoneal region. Table 2 contains details on the treatment modalities.

### 3.5. Outcome

**Figure 2:**
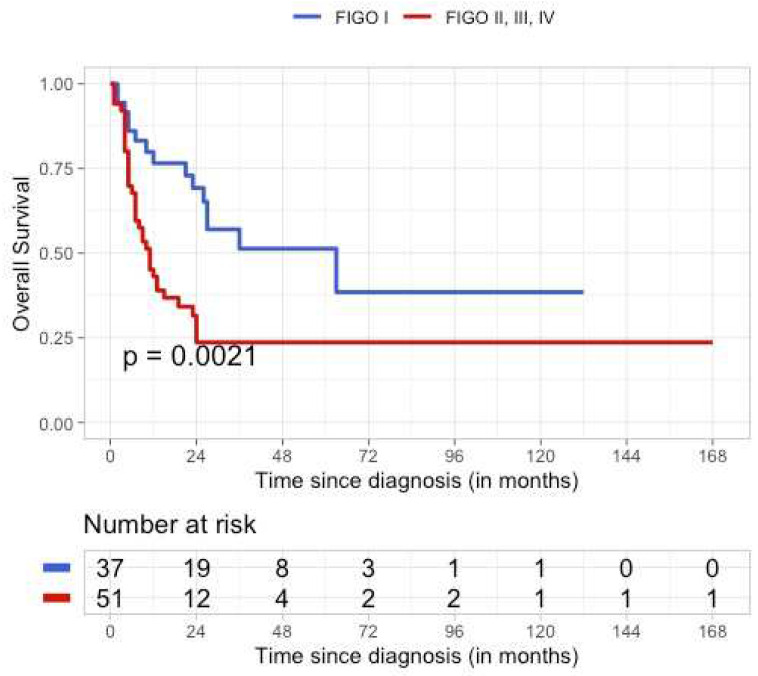
Kaplan–Meier estimates of survival.

The follow-up and outcome were only reported for 88 (29%) patients, with a median follow-up time of 12.5 months (range 1–168 months). Since the number of patients for FIGO stages II, III, and IV was low, we decided to combine these stages as one group in this study. The estimated hazard ratio from the Cox model was equal to 2.530 (95%CI: 1.377; 4.651), with FIGO I as the reference category. FIGO I was classified as early stage, and FIGO II–IV were classified as advanced stage. A total of 37 tumors were FIGO stage I, 12 were stage II, 27 were stage III, and 12 were stage IV. Fifty-one patients died of disease (DOD) (60%). The five-year overall survival (OS) for patients with FIGO stage I was 51% (95% CI: 35–75), and 24% (95%:14–40) for patients with FIGO stages II, III, and IV combined (Figure 2).

Recurrences occurred in 36.4% (32/88) of patients and mainly involved the pelvis and abdomen. The metastatic pattern of SCCOHT involved the lungs, liver, pancreas, bladder, mediastinum, bones, spleen, cerebellar, vagina, and breast in our reported patients.

### 3.6. Potential Targeted Therapies

Fourteen articles provided suggestions for targeted therapies [2,11,34,42,60,64,65,71,73,75,76,77,78]. The majority of the articles discussed the promising results from mainly preclinical studies. These results are thoroughly described in the article by Tischkowitz et al. [11]. They suggest that immunotherapy appears to be the best first choice for treatment. The addition of CDK-4/6 inhibitors and epigenetic therapies also have some beneficial reported outcomes. Table 4 lists potential targeted therapies such as EZH2 inhibitors, PD-1 inhibitors, and CDK-4/6 inhibitors.

## 4. Discussion

SCCOHT is a rare and aggressive ovarian malignancy, presenting mainly in adolescents and young women (range 0–55 years, median age 24 years) [1,2]. Although various treatment approaches have been proposed, there is no international consensus on medical therapy and surveillance [8]. Unfortunately, the outcome of these patients is poor, especially in patients with advanced disease. With this systematic review, we aim to generate an overview of the current knowledge on the incidence, treatment, genetic characteristics, clinical presentation, and survival of patients with SCCOHT.

In this systematic review, we were able to identify 306 patients with SCCOHT reported previously in the literature after 1990, for whom the clinical presentations, genetic characteristics, treatments, and outcomes were described. Unfortunately, the quality of the data was poor, and the follow-up duration was short; high-quality studies with patient and treatment data were not available. As a consequence, this systematic review is mainly based on single case reports and case series. This underlines the need for a prospective collection of diagnostic, treatment, and outcome parameters of young women with SCCOHT. Harmonization and international collaboration are warranted to be able to develop international treatment guidelines to improve the survival chances of young women with SCCOHT.

The median age of the patients presenting with SCCOHT was consistent with previous findings. The clinical presentation was non-specific and similar to cases of more common ovarian malignancies or other more common causes of abdominal pain and distension. One hundred and forty patients were diagnosed at FIGO stage I (45.7%). The other patients were diagnosed with advanced disease, which is comparable to previous data [5,79]. However, we realize that staging was not always adequately reported in the articles [5].

The early diagnosis of ovarian tumors, including SCCOHT, is of crucial importance for optimal treatment outcome. Tumor markers, such as CA-125, can be useful tools that can help to distinguish between benign and malignant ovarian masses. CA-125 levels of less than 35 U/mL are now accepted as normal. Elevated levels of CA-125 are more strongly associated with serous rather than mucinous tumors. It is now widely accepted that the tumor marker CA-125 is a predictive and prognostic factor in CA-125-positive ovarian cancers. The serum CA-125 level is a strong prognostic factor for the overall survival and progression-free survival in ovarian cancer. There is an inverse relationship between serum CA-125 levels and survival in ovarian cancer. That means that a decreasing level generally indicates a positive response to cancer therapy, while an increasing level indicates tumor recurrence and poor survival [80]. Little is known about the prognostic value of CA-125 in patients with SCCOHT. In this systematic review, the CA-125 results were only described for 54 of 306 patients. Of these 54 patients, CA-125 was elevated in 43 (80%). 

In patients with SCCOHT, testing for mutations in the SMARCA4 gene is crucial for an accurate diagnosis. The development of a SCCOHT-specific blood biomarker will definitely aid in early diagnosis, treatment monitoring, and surveillance. 

This review shows a great diversity in therapeutic approaches. Surgery, followed by adjuvant chemotherapy, was found to be the most commonly chosen treatment. The additional value of radiotherapy, HDC with ASCT, or a combination of these treatments is not clear. The optimal surgical approach is unknown, but since unilateral disease is present in most cases, Young et al. suggest that fertility-conserving therapy, a USO, might be preferable in this young patient age group [5]. Other studies also suggest that there may be room for fertility-conserving surgery in the early stages [1,22,31,39,52,55]. Furthermore, Pressey et al. recommend a limited diagnostic procedure followed by an intensive chemotherapy regimen and a second-look operation [2]. The majority of women affected by SCCOHT are of reproductive age; therefore, fertility-conserving surgery appears to be an intriguing alternative. However, given the poor prognosis of this tumor and the small number of cases, there are no recommendations on this subject yet [49].

As shown in this review, all chemotherapeutic regimens were platinum based (either cisplatin or carboplatin). A commonly used regimen was VPCBAE for the first-line treatment of advanced SCCOHT [20]. It should be noted that the VPCBAE regimen is associated with severe toxicities compared to bleomycin, etoposide, and cisplatin (BEP) [55]. Blanc-Durand et al. confirmed the efficacy of the PAVEP regimen in combination with optimal cytoreductive surgery, achieving a complete response for 89% of the patients, particularly for 80% of the patients with residual disease [68]. Also, the role of the inclusion of taxanes in the chemotherapy regimen might be of value [33].

The potential benefit of HDC ASCT in SCCOHT should be kept in mind [2,9]. Blanc-Durand et al. demonstrated promising survival rates in patients treated with HDC ASCT, even for patients with advanced diseases. However, HDC ASCT is also associated with severe morbidity and treatment-related mortality, and thus should be limited to centers with HDC ASCT expertise [68].

Studies describing the use of radiotherapy provide no clear evidence of its role in the treatment of patients with SCCOHT [2,5,30,81]. However, distinguishing the effects of radiotherapy and chemotherapy and their distinct impact on survival is impossible. As a result, the use of radiation and whether it should be given to the pelvis alone, the pelvis and para-aortic area, or the whole abdomen is still unclear.

Several promising new therapies are in various stages of development. The discovery of SMARCA4 loss in 2014 as a primary driver of SCCOHT tumorigenesis has opened the way for developmental therapies that target SWI/SNF complex alterations [2]. There is an urgent need for the development of new (international) treatment guidelines, including targeted therapies, for patients with SCCOHT. Conventional chemotherapy alone will most likely not be the answer for treating this aggressive ovarian cancer. Immunotherapy, most likely in combination with conventional chemotherapy, might be the best choice for first-line treatment in the near future [11].

Our findings show that survival is associated with FIGO stage at diagnosis. However, we were only able to calculate survival in 88/306 patients. The survival of the remaining 218 patients was reported for the cohort as a whole in a different case series. As survival is strongly associated with the FIGO stage at diagnosis, diagnosing SCCOHT at an early stage is critical. Increasing awareness and knowledge about SCCOHT might contribute to the early diagnosis of this rare and aggressive disease. 

Our systematic review is limited by incomplete reports that did not describe the relevant information, such as complete pathology reports, treatment details, and genetic information. Therefore, possible tumor misclassifications cannot be excluded. In addition, the unknown SMARCA4 mutation status prior to 2014 leads to potentially erroneously excluded studies reporting the large cell variant of SCCOHT. The rarity of SCCOHT may have also led to misdiagnosis, resulting in fewer reported patient cases. Moreover, the results mainly consist of single case reports from various centers. Some patients were treated at gynecological cancer treatment centers, while others were treated at peripheral centers, possibly leading to a difference in full surgical staging, which may have contributed to better or poorer outcomes and selection or publication bias. Since the majority of studies are single case reports, which showed much heterogeneity, there was no possibility to perform a quantitative meta-analysis in this review.

Despite these limitations, we believe the findings of this systematic review provide a relevant summary of patient and treatment characteristics, and it shows the heterogeneity of diagnostic modalities employed and the wide variation in treatment approaches in this group of patients. This review also emphasizes the importance of the awareness of this rare diagnosis in young women with an ovarian mass. The discovery of SMARCA4 loss in this type of cancer will help in identifying patients with SCCOHT and might lead to new options for targeted drug therapies. In addition, organoid technology may aid in the development of new targeted therapies. Since SCCOHT is a rare and aggressive type of cancer, the centralization of treatment in tertiary care hospitals will be of benefit for these patients.

## 5. Conclusions

SCCOHT is a rare and aggressive ovarian cancer, with a poor prognosis, and information on adequate treatment for this cancer is lacking. Testing for mutations in the SMARCA4 gene is crucial for an accurate diagnosis and may lead to new treatment options. Harmonization and international collaboration to obtain high-quality data on diagnostic investigations, treatment, and outcome are warranted to be able to develop international treatment guidelines to improve the survival chances of young women with SCCOHT. Since conventional chemotherapy alone may not be sufficient in treating this very aggressive type of ovarian cancer, the possible role of different therapeutic targets for systemic treatment (PD-1, CDK 4-6 inhibitors) should be explored further.

## Figures and Tables

**Table 1:** Patient characteristics.

**Number of Patients**	
N	306
**Age at diagnosis (years)**	
Median Range	25 1–60
**Symptoms (N)**	
Abdominal pain Abdominal swelling General/other symptoms Asymptomatic	172 87 173 3
**Palpable mass at physical examination (N)**	
Palpable pelvic mass No palpable pelvic mass	54 252
**Imaging (N)**	
USS Unspecified Abdominal Transvaginal Mediastinum CT Unspecified Abdominal Pelvic Chest Cerebrum Total body PET MRI Unspecified Abdominal Pelvic Total body X-ray Abdominal Chest Skeletal Scintigraphy Unknown	15 13 8 1 19 24 14 9 2 1 1 1 6 3 1 3 4 6 244
**Tumor laterality (N)**	
Right Left Bilateral Unknown	112 96 5 93
**Hypercalcemia (N)**	
Yes No Unknown	98 94 114
**FIGO Stage (N)**	
I II III IV Unknown	140 19 123 23 1
**Epithelial marker CA-125 (N)**	
Elevated Normal Unknown	43 11 252
**Mutation status (N)**	
Reported SMARCA4 loss	24

**Table 2:** Treatment modalities (*n* = 306).

Surgery	11
Surgery + Chemotherapy	225 *
Surgery + Radiotherapy	Unclear *
Chemotherapy only (palliative treatment)	1
Chemotherapy + Radiotherapy	1
Surgery + Chemotherapy + Radiotherapy	21 *
Surgery + Chemotherapy + HDC ASCT	23
Surgery + Chemotherapy + Radiotherapy + HDC ASCT	23
No treatment	1

HDC ASCT; High-dose chemotherapy with autologous stem cell transplant; * This number is an estimation since in the patient series of Young et al. (1994) [5] the exact number of patients with different treatment modalities was not described in detail.

**Table 3:** Different chemotherapy regimens in the 1st and 2nd line.

**Chemotherapy Regimens**
**Platinum-Paclitaxel-based regimens**
PTax, PETax, CarboTax, CarboTaxE, PE+CarboTax, CarboIfoCtax, CarboE, TaxA
**Platinum-Bleomycin-based regimens** PCBAE, BEP, VPCBAE, BEP MTX AP, VPB,
**Platinum-Ifosfamide-based regimens** PIfoA, CarboEIfoCVCRAct, VCRAC IfoE, VCRACIfoE, CarboIfo, EIfoP, IfoTopo
**Platinum-Doxorubicin-based regimens** PAEC, PAVE, VCR VACPE, PCA
**High-dose chemotherapy** CarboEMel, CarboEC, BuMelThio, CarboE
**Miscellaneous** Doc, Gem, Topo, IriA, Pem, CapP

A: doxorubicin, Act: actinomycin, B: bleomycin, Bu: busulfan, C: cyclphosphamide, Carbo: carboplatin, Cap: Capecitabine Doc: docetaxel, E: etoposide, Gem: gemcitabin, Ifo: ifosfamide, Iri: irinotecan, Mel: melfalan, MTX: methotrexate, P: cisplatin, Pem, Pembrolizumab Tax: paclitaxel, Thio: thiotepa, Topo: topotecan, V: vinblastine, VCR: vincristine.

**Table 4:** Potential targeted therapies.

Medicine	Mechanism
EZH2 inhibitor	Inactivation of SMARCA4 leads to overexpression of the oncogenic activities of EZH2 through transcriptional repression caused by aberrant H3K27me3.
PD-1 inhibitor	SCCOHT tumors express PD-L1 with associated T-cell infiltration, PD-1 and PD-L1 inhibitors may offset adaptive immune evasion of the SCCOHT tumor cells
CDK-4/6 inhibitor	SMARCA4 loss has been shown to lead to downregulation of cyclin D1, limiting the activity of CDK-4/6 and promoting sensitivity to CDK-4/6 inhibitors

EZH2 = Enhancer of zeste homolog 2; H3K27me3 = Histone H3 trimethylated at lysine 27; PD-1 = Programmed Cell Death Protein 1; PD-L1 = Programmed death-ligand 1; CDK-4/6 = cyclin-dependent kinase-4/6.

## Supplementary Materials

The following supporting information can be downloaded at https://www.mdpi.com/article/10.3390/cancers15153794/s1, Table S1: Full search strategy in Pubmed and Embase; Table S2: Quality assessment of the included articles based on the STROBE Statement; Table S3: Overview articles; Figure S1: Relationship between the type of surgery and the FIGO stage.
