# Supplementary material for: Small Cell Carcinoma of the Ovary, Hypercalcemic Type (SCCOHT): Patient Characteristics, Treatment, and Outcome—A Systematic Review
Source: Cancers (Basel). 2023 Jul 26;15(15):3794. doi: 10.3390/cancers15153794 (PMC10417391; doi:10.3390/cancers15153794)
Supplement: Supplementary file 1 [file cancers-15-03794-s001.zip › cancers-2499099-SI.pdf]

Supplementary Table S1: Full search strategy in Pubmed and Embase

**Embase full search** ('small cell carcinoma of the ovary hypercalcemic type'/de OR 'small cell carcinoma of the ovary hypercalcaemic type'/de OR 'small cell carcinomas of the ovary hypercalcemic type'/de OR 'small cell carcinomas of the ovary hypercalcaemic type'/de OR 'ovarian small cell carcinoma of hypercalcemic type'/de OR 'small cell ovarian carcinoma hypercalcemic type'/de OR 'small cell carcinoma of the ovary is classi into the hypercalcemic type'/de OR 'ovary small cell carcinoma hypercalcemic type'/de OR (hypercalcemia/de AND (ovary/exp OR 'ovary tumor'/exp) AND ('small cell carcinoma'/de OR 'small cell ovary carcinoma'/de)) OR ((small-cell* AND ovar* AND (hypercalcem* OR hypercalcaem*)) OR sccoht OR SCC-HT):ab,ti) AND [english]/lim NOT ([animals]/lim NOT [humans]/lim) NOT [conference abstract]/lim AND [1990-2030]/py<br>*Last performed: 12th of December 2022*<br>*Total of 272 results*<br>*Records after duplicates removed: 47*

Medline ALL full search

((Hypercalcemia/ AND (exp Ovary/ OR exp Ovarian Neoplasms/) AND (Carcinoma, Small Cell/)) OR ((small-cell* AND ovar* AND (hypercalcem* OR hypercalcaem*)) OR sccoht OR SCC-HT).ab,ti.) AND english.la. NOT (exp animals/ NOT humans/) NOT (conference abstract) AND 1990:2030.(sa_year).

Last performed: 12th of December 2022

Total of 239 results

Records after duplicates removed: 239

Web of Science Core Collection* full search

TS=(((small-cell* AND ovar* AND (hypercalcem* OR hypercalcaem*)) OR sccoht OR SCC-HT) NOT (animals NOT humans)) AND DT=(Article OR Review OR Letter OR Early Access) AND LA=(English) AND py=(1990-2030)

Last performed: 12th of December 2022

Total of 302 results

Records after duplicates removed: 88

Cochrane Central Register of Controlled Trials full search

(((small NEXT cell* AND ovar* AND (hypercalcem* OR hypercalcaem*)) OR sccoht OR SCC NEXT HT):ab,ti) NOT (animals NOT humans)

Last performed: 12th of December 2022

Total of 1 results

Records after duplicates removed: 0

Supplementary Table S2: Quality assessment of the included articles based on the STROBE Statement

Study	1a	1b	2	3	4	5	6a	6b	7	8	9	10	11	12a	12b	12c	12d	12e	13a	13b	13c	14a	14b	14c	15	16a	16b	16c	17	18	19	20	21	22
**Agaimy** 2015	●	●	●	○	●	◐	◐	○	○	○	○	●	○	○	○	○	○	○	○	○	○	◐	○	●	○	○	○	○	○	●	○	●	○	●
**Agarwal** 2017	●	●	●	○	●	○	○	○	○	○	○	◐	○	○	○	○	○	○	○	○	○	◐	○	●	○	○	○	○	○	●	○	●	○	○
**Aggarwal** 2021	●	●	●	○	●	○	○	○	○	○	○	◐	○	○	○	○	○	○	○	○	○	◐	○	●	○	○	○	○	○	●	○	●	○	○
**Atwi** 2021	●	●	●	○	●	○	○	○	○	○	○	◐	○	○	○	○	○	○	○	○	○	◐	○	○	○	○	○	○	○	●	○	◐	○	○
**Bailey** 2015	●	●	●	○	●	○	○	○	○	○	○	◐	○	○	○	○	○	○	○	○	○	◐	○	●	○	○	○	○	○	●	○	●	○	○
**Barondeau** 2010	●	●	●	○	●	○	○	○	○	○	○	◐	○	○	○	○	○	○	○	○	○	◐	○	●	○	○	○	○	○	◐	○	●	○	○
**Benito** 2015	●	○	●	○	●	○	○	○	○	○	○	◐	○	○	○	○	○	○	○	○	○	◐	○	●	○	○	○	○	○	●	○	●	○	○
**Bitton** 2014	●	●	●	○	●	○	○	○	○	○	○	◐	○	○	○	○	○	○	○	○	○	◐	○	●	○	○	○	○	○	◐	○	●	○	○
**Blanc-Durand** 2020	●	●	●	○	●	●	●	○	○	○	○	●	○	●	○	○	○	●	○	○	○	●	○	●	○	●	○	○	○	●	●	●	○	●
**Chen** 2005	●	●	●	○	◐	○	○	○	○	○	○	◐	○	○	○	○	○	○	○	○	○	◐	○	●	○	○	○	○	○	○	○	◐	○	○
**Chen** 2006	●	●	●	○	●	○	○	○	○	○	○	◐	○	○	○	○	○	○	○	○	○	◐	○	○	○	○	○	○	○	○	○	◐	○	○
**Christin** 2008	●	●	●	○	●	○	○	○	○	○	○	◐	○	○	○	○	○	○	○	○	○	◐	○	●	○	○	○	○	○	●	○	●	○	○
**Connor** 2019	●	●	●	○	●	●	○	○	○	○	○	◐	○	○	○	○	○	○	○	○	○	◐	○	●	○	○	○	○	○	●	○	●	○	●
**David** 2018	●	●	●	○	●	○	○	○	○	○	○	◐	○	○	○	○	○	○	○	○	○	◐	○	●	○	○	○	○	○	●	○	●	○	●
**Distelmaier** 2006	●	●	●	○	●	●	◐	○	○	○	○	●	○	○	○	○	○	○	◐	◐	○	◐	●	●	●	○	○	○	○	●	◐	●	○	●
**Feng** 2020	●	●	●	○	●	○	○	○	○	○	○	◐	○	○	○	○	○	○	○	○	○	◐	○	●	○	○	○	○	○	●	○	●	○	○
**Ghazi** 2017	●	●	●	○	●	○	○	○	○	○	○	◐	○	○	○	○	○	○	○	○	○	◐	○	●	○	○	○	○	○	●	○	●	○	●
**Ghribi** 2014	●	●	●	○	●	○	○	○	○	○	○	◐	○	○	○	○	○	○	○	○	○	◐	○	●	○	○	○	○	○	●	○	●	○	○
**Gupta** 2020	●	●	●	○	●	●	●	○	○	○	○	●	○	○	○	○	○	○	○	○	○	◐	○	●	○	○	○	○	○	●	○	●	○	●
**Hamilton** 2009	●	○	●	○	●	○	○	○	○	○	○	◐	○	○	○	○	○	○	○	○	○	◐	○	◐	○	○	○	○	○	●	○	●	○	○
**Han** 2022	●	●	●	○	●	○	○	○	○	○	○	○	○	○	○	○	○	○	○	○	○	○	○	◐	○	○	○	○	○	●	○	●	○	●
**Harrison** 2006	●	●	●	○	●	●	◐	○	○	○	○	●	○	○	○	○	○	○	◐	◐	○	◐	●	●	●	○	○	○	○	●	◐	●	○	○

Study	1a	1b	2	3	4	5	6a	6b	7	8	9	10	11	12a	12b	12c	12d	12e	13a	13b	13c	14a	14b	14c	15	16a	16b	16c	17	18	19	20	21	22
**Isonishi** 2008	●	●	●	○	●	◐	○	○	○	○	○	◐	○	○	○	○	○	○	○	○	○	◐	○	●	●	○	○	○	○	●	○	●	○	○
**Joseph** 2020	●	●	●	○	●	○	○	○	○	○	○	◐	○	○	○	○	○	○	○	○	○	◐	○	●	○	○	○	○	○	◐	○	◐	○	○
**Kalogeropoulos** 2018	●	●	●	○	●	○	○	○	○	○	○	◐	○	○	○	○	○	○	○	○	○	◐	○	●	○	○	○	○	○	●	○	●	○	○
**Kascak** 2016	●	●	●	○	●	○	○	○	○	○	○	◐	○	○	○	○	○	○	○	○	○	◐	○	●	○	○	○	○	○	●	○	●	○	●
**Khosla** 2018	●	●	●	○	●	○	○	○	○	○	○	◐	○	○	○	○	○	○	○	○	○	◐	○	●	○	○	○	○	○	◐	○	●	○	○
**Lavrut** 2016	●	●	●	○	●	○	○	○	○	○	○	◐	○	○	○	○	○	○	○	○	○	◐	○	●	○	○	○	○	○	●	○	●	○	○
**Li** 2022	●	●	●	○	●	○	○	○	○	○	○	◐	○	○	○	○	○	○	○	○	○	◐	○	●	○	○	○	○	○	●	○	●	○	●
**Mathey** 2019	●	●	●	○	●	○	○	○	○	○	○	◐	○	○	○	○	○	○	○	○	○	◐	○	●	○	○	○	○	○	●	○	●	○	●
**McCormick** 2009	●	●	●	○	●	○	○	○	○	○	○	◐	○	○	○	○	○	○	○	○	○	◐	○	●	○	○	○	○	○	●	◐	●	○	○
**McDonald** 2012	●	●	●	○	●	○	○	○	○	○	○	◐	○	○	○	○	○	○	○	○	○	◐	○	◐	○	○	○	○	○	●	○	●	○	○
**Megank** 1998	●	●	●	○	●	○	○	○	○	○	○	◐	○	○	○	○	○	○	○	○	○	◐	○	●	○	○	○	○	○	◐	○	●	○	○
**Milena** 2015	●	●	●	○	●	○	○	○	○	○	○	◐	○	○	○	○	○	○	○	○	○	◐	○	●	○	○	○	○	○	●	○	●	○	○
**Montalto** 2011	●	○	●	○	●	○	○	○	○	○	○	◐	○	○	○	○	○	○	○	○	○	◐	○	●	○	○	○	○	○	●	○	●	○	○
**Montebello** 2021	●	●	●	○	●	○	○	○	○	○	○	◐	○	○	○	○	○	○	○	○	○	◐	○	○	○	○	○	○	○	●	○	●	○	●
**Najib** 2017	●	●	●	○	●	○	○	○	○	○	○	◐	○	○	○	○	○	○	○	○	○	◐	○	●	○	○	○	○	○	○	○	●	○	○
**Origori** 2013	●	●	●	○	●	○	○	○	○	○	○	◐	○	○	○	○	○	○	○	○	○	◐	○	●	○	○	○	○	○	●	○	●	○	●
**Peccatori** 1993	●	●	●	○	●	○	○	○	○	○	○	●	○	○	○	○	○	○	○	○	○	◐	○	●	○	○	○	○	○	●	○	●	○	○
**Peter** 2000	●	●	●	○	●	○	○	○	○	○	○	◐	○	○	○	○	○	○	○	○	○	◐	○	◐	○	○	○	○	○	◐	○	●	○	○
**Qin** 2018	●	●	●	○	●	○	○	○	○	○	○	◐	○	○	○	○	○	○	○	○	○	◐	○	●	○	○	○	○	○	●	○	●	○	●
**Rasmussen** 1991	●	●	○	○	●	○	○	○	○	○	○	◐	○	○	○	○	○	○	○	○	○	◐	○	●	○	○	○	○	○	●	○	●	○	○
**Reed** 1994	●	●	◐	○	●	○	○	○	○	○	○	◐	○	○	○	○	○	○	○	○	○	◐	○	●	○	○	○	○	○	●	○	●	○	○
**Reyes-Tobar** 2021	●	●	●	○	●	○	○	○	○	○	○	◐	○	○	○	○	○	○	○	○	○	◐	○	●	○	○	○	○	○	●	○	●	○	○
**Rovithi** 2011	●	●	●	○	●	○	○	○	○	○	○	◐	○	○	○	○	○	○	○	○	○	◐	○	●	○	○	○	○	○	●	○	●	○	○

Study	1a	1b	2	3	4	5	6a	6b	7	8	9	10	11	12a	12b	12c	12d	12e	13a	13b	13c	14a	14b	14c	15	16a	16b	16c	17	18	19	20	21	22
**Sahay** 2020	●	●	●	○	●	○	○	○	○	○	○	◐	○	○	○	○	○	○	○	○	○	◐	○	●	○	○	○	○	○	●	○	●	○	●
**Sanders** 2022	●	●	●	○	●	○	○	○	○	○	○	◐	○	○	○	○	○	○	○	○	○	◐	○	●	○	○	○	○	○	●	○	●	○	●
**Sassi** 2007	●	●	●	○	●	○	○	○	○	○	○	◐	○	○	○	○	○	○	○	○	○	◐	○	○	○	○	○	○	○	◐	○	●	○	○
**Schleef** 1999	●	●	●	○	●	○	○	○	○	○	○	◐	○	○	○	○	○	○	○	○	○	◐	○	●	○	○	○	○	○	◐	○	●	○	○
**Schweiger** 2002	◐	○	○	○	●	○	○	○	○	○	○	◐	○	○	○	○	○	○	○	○	○	◐	○	●	○	○	○	○	○	●	○	●	○	○
**Selvaggi** 1994	●	●	●	○	●	○	○	○	○	○	○	◐	○	○	○	○	○	○	○	○	○	◐	○	●	○	○	○	○	○	●	○	●	○	○
**Sholler** 2005	●	●	●	○	●	○	○	○	○	○	○	◐	○	○	○	○	○	○	○	○	○	◐	○	●	○	○	○	○	○	●	○	●	○	○
**Shrimali** 2010	●	●	●	○	●	○	○	○	○	○	○	◐	○	○	○	○	○	○	○	○	○	◐	○	●	○	○	○	○	○	●	○	●	○	○
**Simões** 2022	●	●	●	○	●	○	○	○	○	○	○	◐	○	○	○	○	○	○	○	○	○	◐	○	●	○	○	○	○	○	●	○	●	○	●
**Stephens** 2012	●	●	●	○	●	○	○	○	○	○	○	◐	○	○	○	○	○	○	○	○	○	◐	○	●	○	○	○	○	○	○	○	●	○	○
**Stewart** 2016	●	●	●	○	●	○	○	○	○	○	○	◐	○	○	○	○	○	○	○	○	○	◐	○	●	○	○	○	○	○	●	○	●	○	○
**Taraszewski** 1990	●	●	●	○	●	○	○	○	○	○	○	◐	○	○	○	○	○	○	○	○	○	◐	○	●	○	○	○	○	○	●	○	●	○	○
**Tewari** 1997	●	●	●	○	●	○	○	○	○	○	○	◐	○	○	○	○	○	○	○	○	○	◐	○	●	○	○	○	○	○	●	○	●	○	○
**Vivod** 2021	●	●	●	○	●	○	○	○	○	○	○	◐	○	○	○	○	○	○	○	○	○	◐	○	●	○	○	○	○	○	●	○	●	○	○
**Wallbillich** 2012	●	○	●	○	●	○	○	○	○	○	○	◐	○	○	○	○	○	○	○	○	○	◐	○	●	○	○	○	○	○	●	○	●	○	○
**Woopen** 2012	●	●	●	○	●	●	●	○	○	○	○	●	○	○	○	○	○	○	○	○	○	◐	●	●	●	○	○	○	○	●	○	●	○	○
**Wynn** 2004	●	●	●	○	●	○	○	○	○	○	○	◐	○	○	○	○	○	○	○	○	○	◐	○	●	○	○	○	○	○	◐	○	●	○	○
**Yoshida** 2018	●	○	●	○	●	○	○	○	○	○	○	◐	○	○	○	○	○	○	○	○	○	◐	○	●	○	○	○	○	○	●	○	●	○	○
**Young** 1994	●	●	●	○	●	●	●	○	○	○	○	●	○	○	○	○	○	○	◐	◐	○	○	●	●	●	○	○	○	○	●	○	●	○	○
**Young** 2010	●	○	○	○	●	○	○	○	○	○	○	◐	○	○	○	○	○	○	○	○	○	●	○	●	○	○	○	○	○	○	○	○	○	○
**Zagouri** 2012	●	○	●	○	●	○	○	○	○	○	○	◐	○	○	○	○	○	○	○	○	○	◐	○	●	○	○	○	○	○	○	○	◐	○	○
**Zaied** 2012	●	●	●	○	●	○	○	○	○	○	○	◐	○	○	○	○	○	○	○	○	○	◐	○	●	○	○	○	○	○	◐	○	●	○	○

● = present; ○ = absent; ◐ = unclear/incomplete

STROBE Statement – Checklist of items that should be included in report of observational studies

Title and abstract;

1. Title and abstract
    a. Indicate the study's design with a commonly used term in the title or the abstract
    b. Provide in the abstract an informative and balanced summary of what was done and what was found

Introduction

2. Background/rationale: explain the scientific background and rationale for the investigation being reported
3. Objectives: state specific objectives, including any prespecified hypothesis

Methods

4. Study design: present key element of study design early in the paper
5. Setting: describe the setting, locations, and relevant dates, including periods of recruitment, exposure, follow-up, and data-collection
6. Participants:
    a. Give the eligibility criteria and the sources and methods of selection of participants. Describe methods of follow-up
    b. For matched studies, give matching criteria and number of exposed and unexposed
7. Variables: clearly define all outcomes, exposures, predictors, potential confounders, and effect modifiers. Give diagnostic criteria, if applicable
8. Data sources/measurement: for each variable of interest, give sources of data and details of methods of assessment (measurement). Describe comparability of assessment methods if there is more than one group
9. Bias: describe any efforts to address potential sources of bias
10. Study size: explain how the study size was arrived at
11. Quantitative variables: explain how quantitative variables were handled in the analyses. If applicable, describe which grouping were chosen and why
12. Statistical methods;
    a. Describe all statistical methods, including those used to control for confounding

b. Describe any methods used to examine subgroups and interactions

c. Explain how missing data were addressed

d. If applicable, explain how loss to follow-up was addressed

e. Describe any sensitivity analyses

Results

13. Participants;

    a. Report numbers of individuals at each stage of study – eg numbers potentially eligible, examined for eligibility, confirmed eligible, included in the study, completing follow-up, and analysed

    b. Give reasons for non-participating at each stage

    c. Consider use of a flow diagram

14. Descriptive data;

    a. Give characteristics of study participants (eg demographic, clinical, social) and information on exposures and potential confounders

    b. Indicate number of participants with missing data for each variable of interest

    c. Summarise follow-up time (eg, average and total amount)

15. Outcome data: report numbers of outcome events or summary measures over time

16. Main results;

    a. Give unadjusted estimates and, if applicable, confounder-adjusted estimates and their precision (eg, 95% confidence interval). Make clear which confounders were adjusted for and why they were included

    b. Report category boundaries when continuous variable were categorized

    c. If relevant, consider translating estimates of relative risk into absolute risk for a meaningful time period

17. Other analyses: report other analyses done – eg analyses of subgroups and interactions, and sensitivity analyses

Discussion

18. Key results: summarise key results with reference to study objectives

19. Limitations: discuss limitations of the study, taking into account sources of potential bias or imprecision. Discuss both direction and magnitude of any potential bias

20. Interpretation: give a cautious overall interpretation of results considering objectives, limitations, multiplicity of analyses, results from similar studies, and other relevant evidence

21. Generalizability: discuss the generalizability (external validity) of the study results

Other information

22. Funding: give the source of funding and the role of the funders for the present study and, if applicable, for the original study on which the present article is based

Supplementary Table S3: Overview articles

Study	Country	n	Mean age (yrs)	Localization (n)	Hyper-calcemia (n)	Epithelial Tumor Markers (n)	Surgery (n)	FIGO Stage (n)	Chemotherapy regimens (n)	SCT (n)	RT (n)	Genetics	Recurrence (n)	FU (median, months)	Survival (n)
Agaimy 2015 [53]	Germany	3	35	L(1), R(2)	N(1), U(2)	U(3)	TAHBSO(3)	I(1), III(2)	Platinum-based	N(3)	Y(1), N(2)	SMARCA4 loss	Y(2), N(1)	6	A(1), D(2)
Agarwal 2017 [58]	India	1	45	L	N	U	TAHBSO	I	Platinum-Bleomycin-based	N	N	NP	N	U	A
Aggarwal 2021 [72]	India	1	10	U	U	U	USO	III	Platinum-ifosfamide-based	N	N	SMARCA4 loss	N	7	D
Atwi 2021 [73]	USA	1	1	L	Y	U	USO	I	U	N	N	SMARCA4 loss	N	U	A
Bailey 2015 [52]	UK	1	15	L	Y	CA-125+	USO	I	Platinum-ifosfamide-based	Y	N	SMARCA4 loss	N	9	A
Barondeau 2010 [37]	USA	1	16	R	Y	CA-125+ CA 19-9=	USO	IV	Various regimens	N	N	NP	Y	13	D
Benito 2015 [50]	Spain	1	31	R	N	CA-125+	USO	III	NP	N	N	NP	N	5	D
Bitton 2014 [48]	Brazil	1	47	R	N	U	NP	IV	Platinum-based	N	N	NP	N	2	D
Blanc-Durand 2020 [68]	France	44	33	U(44)	Y(6), N(24), U(14)	U(44)	U(44)	I(14), III(21), IV(9)	Platinum-doxorubicin-based(44)	Y(30), N(14)	Y(21) (pelvis), N(23)	NP	Y(26), N(18)	53	A(26), D(18)
Chen 2006 [28]	USA	1	26	R	Y	CA-125+	USO	I	U	N	N	NP	U	U	U
Chen 2005 [26]	USA	1	37	R	Y	CA-125+	TAHBSO	I	Platinum-Paclitaxel-based	N	N	NP	N	27	A
Christin 2008 [32]	France	1	12	L	N	CA-125+ CA 15-3=	USO+ TAHBSO	III	Various regimens	Y	N	NP	Y	168	A
Connor 2019 [64]	USA	1	31	L	N	U	USO	III	Various regimens	N	N	SMARCA4 loss	N	6	D
David 2018 [61]	USA	1	12	L	N	CA-125+	U	I	Various regimens	Y	N	SMARCA4 loss	Y	14	D
Distelmaier 2006 [29]	Germany	9	15	U(9)	Y(4), N(5)	U(9)	USO(9)	I(6), III(3)	Various regimens(9)	Y(3), N(6)	N(9)	NP	Y(6), N(9)	18	A(5), D(4)
Feng 2020 [66]	China	1	21	L	N	CA-125+	TAHBSO	III	NP	N	N	SMARCA4 loss	N	5	D
Ferrera 2000 [23]	USA	1	15	U	N	CA-125+	USO	IV	Platinum-ifosfamide-based	Y	N	NP	U	U	A
Ghazi 2016 [54]	Saudi Arabia	1	35	U	Y	CA-125+	NP	IV	NP	N	N	NP	N	1	D

						CA 19.9=<br>CA 15.3=									
Ghribi 2014 [49]	Tunisia	1	10	R	Y	CA-125=	USO	I	Platinum-Bleomycin-based	N	N	NP	N	84	A
Gupta 2020 [67]	India	3	22	L(2), R(1)	U(3)	U(3)	USO	IV	Various regimens(3)	N(3)	N(3)	SMARCA4 loss	N	4	D(3)
Hamilton 2009 [35]	UK	3	28	L(2), B(1)	N(1), U(2)	CA-125+ (1), = (1), U(1)	USO+TAHBSO(2), TAHBSO(1)	I(1), II (1), III(1)	Various regimens(3)	N(3)	N(3)	NP	Y	4	D(3)
Han 2022 [78]	China	3	28	L(1), R(1), U(1)	N(1), U(2)	CA-125= (1), +(1), U(1)	USO(2), TAHBSO(1)	III(2), IV(1)	Various regimens(2), Platinum-based	N(3)	N(3)	SMARCA4 loss, NP(2)	N(3)	14	A(2), D(1)
Harrison 2006 [30]	Australia, Canada, Europe	17	35	U(17)	Y(4), N(6), U(7)	U(17)	USO+TAHBSO(8), Limited surgery (8), U(1)	I(10), III(6), U(1)	Various regimens(17)	N(17)	Y(7) (para-aortic, whole abdomen, pelvis), N(10)	NP	Y(8), N(9)	13	A(10), D(7)
Ilic 2015 [51]	Serbia	1	60	R	Y	U	TAHBSO	I	U	N	Y (unknown)	NP	N	26	D
Isonishi 2008 [33]	Japan	3	29	L(1), R (1), U(1)	N(2), U(1)	CA-125+(2), U(1)	USO(2), TAHBSO(1)	II(2), III(1)	Varioius regimens(3)	N(3)	Y(1) (whole abdomen), N(2)	NP	Y(3)	5	A(1), D(2)
Kalogeropoulos 2018 [62]	Greece	1	49	L	Y	CA-125+	U	III	Platinum-based	N	N	SMARCA4 loss	N	4	A
Kascak 2016 [56]	Slovakia	1	24	R	Y	CA-125+	USO	I	Platinum-based	N	N	p53 positive	Y	10	D
Khosla 2018 [63]	India	1	16	R	N	U	USO	IV	Various regimens	N	N	SMARCA4 loss	N	24	D
Lavrut 2016 [57]	France	1	14	B	Y	CA-125+	USO+TAHBSO	IV	Platinum- Doxorubicin-based	N	N	SMARCA4 loss	N	4	D
Li 2022 [76]	China	1	36	U	N	CA-125=	USO	I	Various regimens	N	Y	SMARCA4 loss	Y	44	A
Mathey 2019 [65]	Switzerland	1	22	L	N	CA-125+<br>CA 19-9+	USO	I	Platinum-Doxorubicin-based	Y	N	SMARCA4 loss	N	30	A

McCormick 2009 [36]	USA	1	22	L	Y	U	USO+TAHBSO	II	Platinum-based	N	N	NP	Y	10	D
McDonald 2012 [42]	USA	2	17	L(1), R(1)	Y(2)	CA-125+(2)	USO(2)	I(1), II(1)	Various regimens	N(2)	Y(2) (pelvis, whole abdomen)	NP	Y(2)	19	D(2)
Meganck 1998 [21]	Belgium	1	34	R	N	CA-125+	TAHBSO	IV	Platinum-Bleomycin-based	N	N	p53, Ki67 positive	N	5	D
Montalto 2011 [39]	UK	1	17	R	Y	CA-125+	USO	I	Platinum-based	N	N	NP	N	49	A
Montebello 2021 [70]	Malta	1	37	L	Y	CA-125=	TAHBSO	I	Platinum-based	N	Y (pelvis)	NP	N	U	A
Najib 2017 [59]	Iran	1	37	L	N	U	USO+TAHBSO	I	Platinum-based	N	N	NP	N	2	D
Origoni 2013 [47]	Italy	3	34	L(1), R(1), B(1)	Y(2), U (1)	CA-125+(2), U(1)	USO+TAHBSO(2), USO(1)	II(3)	Various regimens(3)	N(3)	N(3)	NP	N(3)	5	D(3)
Peccatori 1993 [17]	Italy	4	23	L(1), R(2), B(1)	Y(1), N(1), U(2)	CA-125+(1), = (1), U(2)	USO(3), TAHBSO(1)	I(2), III(1), IV(1)	Various regimens(4)	Y(1), N(3)	Y(1) (pelvis), N(3)	NP	Y(1), N(3)	15	A(1), D(3)
Pressey 2020 [2]	USA	1	32	R	N	CA-125+ CA19-9=	USO	I	Platinum-Bleomycin-based	Y	N	SMARCA4 loss	N	5	A
Qin 2018 [60]	USA	1	19	L	Y	CA-125+	USO	III	Platinum-Doxorubicin-based	Y	Y (pelvis, para-aortic)	NP	N	96	A
Rasmussen 1991 [16]	Denmark	1	22	R	Y	CA-125=	USO+TAHBSO	I	Platinum-Bleomycin-based	N	Y (para-aortic)	NP	Y	23	D
Reed 1994 [18]	USA	1	31	L	Y	U	TAHBSO	I	Platinum-Bleomycin-based	N	N	NP	N	60	A
Reyes-Tobar 2021 [71]	Chile	1	22	R	U	U	USO+TAHBSO	III	Platinum-based	N	Y	NP	N	30	A
Rovithi 2011 [40]	Greece	1	19	R	Y	CA-125+	TAHBSO	III	Various regimens	N	Y	p53 positive	Y	7	D
Sahay 2020 [69]	India	2	27	L(1), R(1)	Y(1), U(1)	CA-125+(2),	USO+TAHBSO(2)	III(2)	Various regimens(2)	N(2)	N(2)	SMARCA4 loss	N(2)	4	D(2)

						CA19-9= (2)									
Sanders 2022 [77]	USA	1	35	R	N	CA-125+	TAHBSO	III	Platinum-Doxorubicin-Based	Y	N	SMARCA4 loss	N	17	A
Sassi 2007 [31]	Tunisia	1	10	R	N	U	USO	I	Platinum-Bleomycin-based	N	N	NP	N	24	A
Schleef 1999 [22]	Germany	1	8	L	Y	CA-125, CA 19-9, CA 72-4=	USO	I	Various regimens	Y	N	NP	N	9	A
Schweiger 2002 [24]	USA	1	13	R	Y	CA-125=	USO+TAHBSO	I	Various regimens	N	N	NP	Y	36	D
Selvaggi 1994 [19]	USA	1	30	L	N	U	TAHBSO	III	Platinum-based	N	N	NP	N	2	D
Sholler 2005 [27]	USA	1	11	L	N	CA-125+	USO	III	Platinum-Bleomycin-based	N	N	NP	N	30	A
Shrimali 2010 [38]	UK	2	56	L(1), U(1)	N(1), U(1)	CA-125+(2)	NP(1), U(1)	III(1), IV(1)	Various regimens(2)	N(2)	Y(1) (whole brain), N(1)	NP	Y(2)	23	A(1), D(1)
Simões 2022 [75]	Brazil	1	19	R	N	U	USO	IV	Various regimens	Y	N	SMARCB1 loss	N	11	D
Stephens 2012 [41]	USA	1	21	L	U	CA-125+	USO+TAHBSO	III	Various regimens	N	Y (pelvis)	NP	Y	12	D
Stewart 2016 [55]	USA	1	14	L	Y	CA-125+	USO	I	Platinum-Bleomycin-based	N	N	NP	N	132	A
Taraszewski 1990 [15]	USA	1	29	R	Y	U	TAHBSO	II	Platinum-Doxorubicin-based	Y	N	NP	Y	19	D
Tewari 1997 [20]	USA	1	26	R	N	CA-125=	USO+TAHBSO	III	Platinum-Bleomycin-based	N	N	NP	N	66	A
Vivod 2021 [74]	Slovenia	1	31	L	Y	CA-125, CA 15-3, CA 19-9, CA 72-4=	USO	I	Platinum-Paclitaxel-based	N	Y	SMARCA4 loss	Y	27	D
Wallbillich 2012 [43]	USA	3	26	L(2), R(1)	U(3)	U(3)	USO(2), USO+TAHBSO(1)	I(3)	Various regimens(3)	N(3)	N(3)	NP	N(3)	16	A(3)

Woopen 2012 [44]	Germany	4	25	R(3), B(1)	Y(2), N(1), U(1)	CA-125+(3), U(1)	USO(3), TAHBSO(1)	I(2), II(1), III(1)	Various regimens (4)	Y(1), N(3)	N(4)	NP	Y(1), N(3)	22	A(3), D(1)
Wynn 2004 [25]	USA	1	27	R	Y	U	USO+TAHBSO	III	Platinum-Paclitaxel-based	N	N	NP	Y	16	A
Yoshida 2008 [34]	Japan	1	33	R	Y	CA-125+	USO	III	Various regimens	N	Y	SMARCA4 loss	N	7	D
Young 2010 [1]	USA	1	22	R	Y	CA-125+	USO	I	Platinum-Paclitaxel-based	N	Y (whole abdomen)	NP	N	24	A
Young 1994 [5]	USA	150	24	L(60), R(74), U(16)	Y(49), N(30), U(71)	U(150)	USO(69), TAHBSO(45, other(11), U (25)	I(75), II(8), III(65), IV(2)	Platinum-Doxorubicin-based(150)	N(150)	U(150)	NP	U(150)	U	A(60), D(90)
Zagouri 2012 [46]	Greece	1	19	L	Y	CA-125+	USO	II	Various regimens	N	N	p53 positive	N	8	D
Zaied 2012 [45]	Tunisia	1	25	L	N	CA-125+	TAHBSO	I	Platinum-Bleomycin-based	N	N	NP	N	2	D

n= population size; L= left; R= right; B= bilateral; U= unknown; Y= yes; N= no; += elevated; -= normal; CA-125= cancer antigen 125; CA 19-9= cancer antigen (19-9); CA 15-3= cancer antigen 15-3; CA 72-4= cancer antigen 72-4; USO= unilateral salpingo-oophorectomy; TAHBSO= total abdominal hysterectomy with bilateral salpingo-oophorectomy; FU= follow-up; NP= not performed; A= alive; D= dead

Supplementary Figure S1: Relationship between the type of surgery and the FIGO stage.

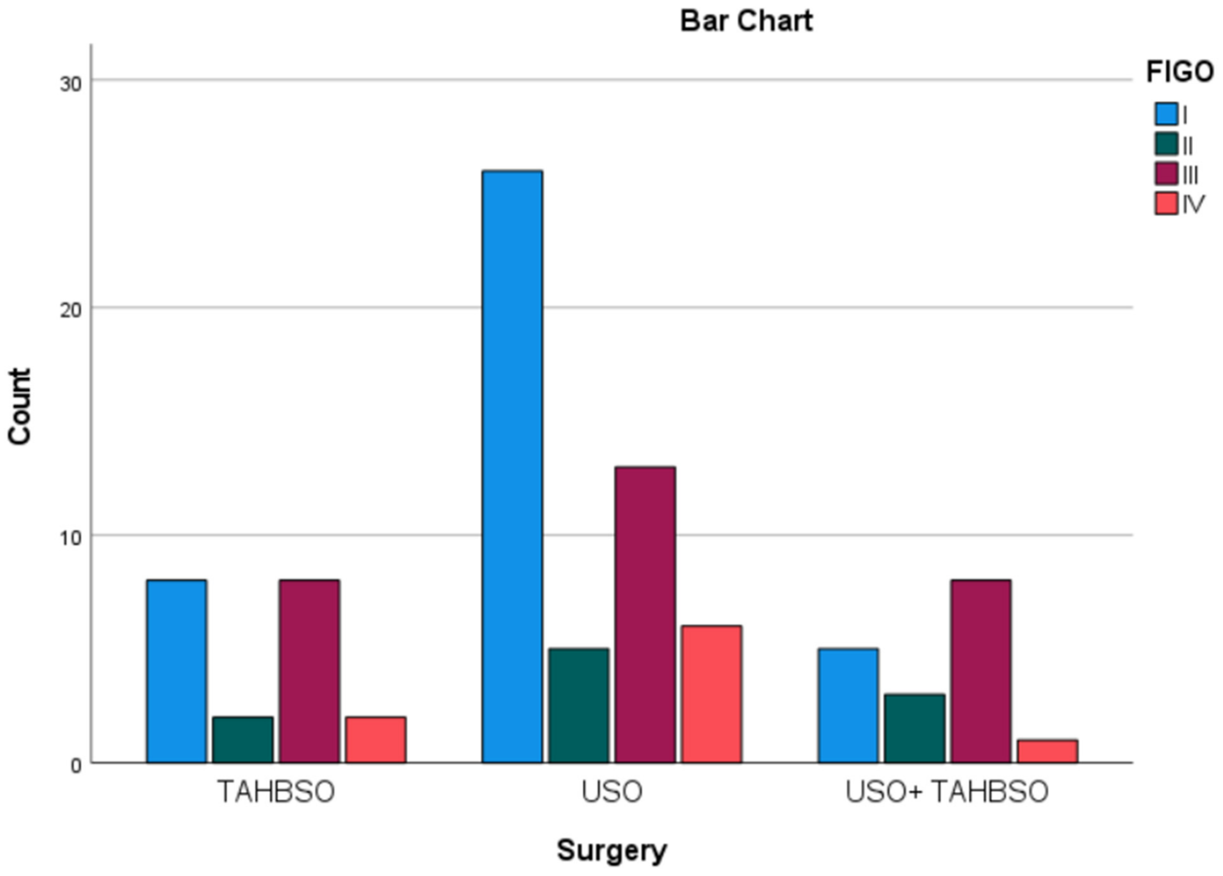

Pearson Chi-Square; 4.771; df= 6; P= 0.574; n= 88

USO= unilateral salpingo-oophorectomy; TAHBSO= total abdominal hysterectomy with bilateral salpingo-oophorectomy
